# Vehicle Trajectory Prediction Method Based on License Plate Information Obtained from Video-Imaging Detectors in Urban Road Environment

**DOI:** 10.3390/s20051258

**Published:** 2020-02-25

**Authors:** Zheng Zhang, Haiqing Liu, Laxmisha Rai, Siyi Zhang

**Affiliations:** 1 College of Transportation, Shandong University of Science and Technology, Qingdao 266000, China; zheng_zhang163@163.com (Z.Z.); ZSY033186ZSY@163.com (S.Z.); 2 College of Electronic and Information Engineering, Shandong University of Science and Technology, Qingdao 266000, China; laxmisha@ieee.org

**Keywords:** vehicle trajectory prediction, license plate data, trip chain, turning state transit

## Abstract

The vehicle license plate data obtained from video-imaging detectors contains a huge volume of information of vehicle trip rules and driving behavior characteristics. In this paper, a real-time vehicle trajectory prediction method is proposed based on historical trip rules extracted from vehicle license plate data in an urban road environment. Using the driving status information at intersections, the vehicle trip chain is acquired on the basis of the topologic graph of the road network and channelization of intersections. In order to obtain an integral and continuous trip chain in cases where data is missing in the original vehicle license plate, a trip chain compensation method based on the Dijkstra algorithm is presented. Moreover, the turning state transition matrix which is used to describe the turning probability of a vehicle when it passes a certain intersection is calculated by a massive volume of historical trip chain data. Finally, a *k*-step vehicle trajectory prediction model is proposed to obtain the maximum possibility of downstream intersections. The overall method is thoroughly tested and demonstrated in a realistic road traffic scenario with actual vehicle license plate data. The results show that vehicles can reach an average accuracy of 0.72 for one-step prediction when there are only 200 historical training data samples. The proposed method presents significant performance in trajectory prediction.

## 1. Introduction

With economic development and the continuous expansion of the scale of cities, the number of vehicles increases sharply, inducing a frequent occurrence of road traffic offenses. In the urban traffic system, the supervision of abnormal vehicles, such as fake license plates, suspected of cases of illegal operation and other anomalies, have been viewed with high precaution by the traffic administration since they seriously threaten normal traffic order and safety. Intelligence analysis about these abnormal vehicles makes broader sense for assisting effective vehicle monitoring and management. The intelligence information includes all the holographic states of a vehicle, for example, the basic data of the vehicle and driver, the trajectory, the origin destination (OD) characteristics, aggregation with other vehicles, and others. It is really an important research topic on the real-time intelligence information analysis method by current devices deployed onboard or roadside.

In many intelligence information types mentioned above, the trajectory of the vehicle contains abundant spatial and temporal distribution features. Using a geographic information system (GIS), the trajectory can be accurately projected on an electronic map to give an intuitive presentation of the vehicle’s movement. Moreover, through the analysis of massive trajectory data, the trip rules of vehicles, such as the OD, and the travel preferences of the driver can also be obtained. These are very valuable for intelligent macro traffic management. In some microscopic traffic control applications, the vehicle trajectory is also meaningful. For example, the single trajectory prediction can be used to provide support for capturing illegal vehicles [1,2], while multiple trajectories prediction can be used to evaluate the short-time traffic volume for designing a proper traffic signal plan [3,4,5,6,7,8].

Using the electronic surveillance cameras deployed on roads, the driving status of a vehicle such as the time, license plate number, speed, lane and direction can all be acquired. On the basis of the road network topology, a vehicle passes through a series of intersections and the trajectory of the vehicle can be obtained easily from the driving status data collected by a video-imaging detector at the intersections. Compared with the methods based on location-based-services (LBS) [9,10,11,12], wireless fidelity (WiFi) probes [13,14,15] and cellular signaling [16,17,18,19], vehicle trajectory extracted form vehicle license plate data has certain advantages in terms of wide adaptability, accuracy and visualization effects. Fully using the driving status acquired by the video-imaging detectors, this paper studies the vehicle trajectory prediction scheme based on the latent travelling rules extracted from massive vehicle license plate data. For a certain vehicle, the turning characteristic at an intersection is described by a turning probability transition matrix in which the probability is calculated according to statistics of historical trip chains from the vehicle license plate. The experimental study shows that the proposed method presents a better performance in a short-term trajectory prediction. 

The rest of the paper is organized as follows. Section 2 presents related work. In Section 3, trip chain building and compensating methods based on vehicle license plates are presented. In Section 4, the turning state transition matrix based on historical trip chains is proposed for trajectory prediction and the one/*k*-step trajectory prediction models are described. The experimental study and the evaluation results are presented in Section 5. In Section 5, we conclude the paper and provide directions for future work.

## 2. Related Work

Vehicle trajectory is important intelligence information for both urban macroscopic traffic management and microscopic traffic control. Generally, vehicle trajectory uses a vehicle’s or driver’s location and identity as major data foundations. According to different vehicle or driver locations and identity acquisition types in an urban traffic environment, the current trajectory-building methods can be classified into the following categories: LBS-based methods, WiFi probe-based methods, cellular signaling-based methods and video-imaging-based methods.

The LBS-based method mainly uses global positioning system (GPS) floating vehicle data to track the target vehicle. The movement of the vehicle is detected continuously in time and the trajectory can be presented visually combined with the application of an electronic map. In [9], based on the vehicles’ historical trips, the relationship between different road segments are built by transforming the road network model into a matrix, and the driving regularity of vehicles is analyzed for the design of the algorithm of vehicle route prediction. In [10], the historical vehicle GPS data is used to match the current trajectories and infer future possible destinations. The method predicts the trajectory by a systematic procedure for describing the features of the similarity of trajectories and destinations. The method also takes the station correlations and the user historical destinations into account. The positive prediction rate of the proposed method can reach 92% under the condition that the test trip has been completed over 70%. In [11], authors propose a vehicle trajectory prediction method based on the hidden Markov model. The relevant parameters are analyzed by historical vehicle GPS data, and the Viterbi algorithm is used to seek the double layers hidden states sequences corresponding to the recent driven trajectory. The future vehicle trajectory is predicted by a novel algorithm based on the hidden Markov model of double-layer hidden states. In [12], using the GPS data, a vehicle trajectory prediction method based on a variable-order Markov model is proposed. Kernel smoothing which combines sequence analysis with the Markov statistic is used for model building. The method presents a higher performance in prediction accuracy. However, these methods are only suitable for some special commercial vehicles which are equipped with GPS or other onboard positioning devices, and not applicable for most private vehicles.

By installing certain WiFi probe devices [13] at intersections or roads, the WiFi probe-based method generates the trajectory by detecting the passing time and the media access control (MAC) addresses of electronic terminals with WiFi-connecting function, such as the onboard unit and driver’s mobile phone. In [14], authors use a WiFi probe as the data collector to scan the mobile devices within a certain range in a certain period of time to obtain the MAC address, reference distance, time stamp and other information of mobile phone. Furthermore, a customer flow prediction model based on seasonal auto regressive integrated moving average (SARIMA) model and BP neural network model is built. In [15], an urban mobility trajectory analysis model based on large-scale WiFi probe request data is built. Unique entries per access point and per hour of WiFi data are aggregated to approximate local population counts by type of user. In the model, spatial network analysis is used to apply the results to the road and pedestrian sidewalk network to identify usage intensity levels and trajectories for individual street segments. The research demonstrates the significant potential in the use of WiFi probe request data for understanding mobility patterns. Similar in [16], authors design a user feature space in which frequent trajectory patterns are used to represent each user as a feature vector based on the anonymized WiFi scan lists. As per the popularity of electronic terminals, the application of WiFi probe is more adaptable than the LBS-based method. However, the identity of the target is denoted by the MAC addresses of the electronic terminal. It is not directly relevant to the vehicle itself. Besides, in order to achieve an urban-wide trajectory analysis, several WiFi probe devices should be built, inducing high expense for construction.

As with the WiFi probe scheme, the cellular signaling-based method also uses the location and identity of electronic terminals to generate the trajectory. By contrast with the former, the location is detected by the mobile station and the identity is generally the mobile user or the MAC of the mobile terminal. For example, in [17], the authors propose a data-driven method for dynamic people-flow prediction based on cellular probe data. The grid-based data transformation and data integration module is proposed to integrate multiple data sources for human daily trajectory generation. Moreover, a dynamic people-flow prediction model based on random forest is also presented. The experimental results show that the proposed method can provide prediction precision of 76.8% and 70% for outbound and inbound people, which is better than the single-feature model. In [18], the authors introduce a mobility modeling method based on real traffic data collected from 4G cellular networks, including data collection, trajectory construction, data noise removal, data storage and analysis. The experiments discover the user’s mobility features, changing of city hotspots, and mobility patterns. However, locating using a mobile cellular network and mobile base station is inaccurate outdoors without supplementary GPS or WiFi devices. Hence, the precision of these methods is relatively low and they are only suitable for microscopic traffic and population evolution analysis.

Fully using the driving status data collected by electronic surveillance cameras on roads, the vehicle trajectory is acquired by time detecting and license plate number series. In [19], an offline method for historical OD pattern estimation based on automatic license plate recognition data is proposed. A particle filter is used to estimate the probability of a vehicle trajectory from all possible candidate trajectories combined with the time geography theory. In this method, the path flow estimation process is conducted through dividing the reconstructed complete trajectories of all detected vehicles into multiple trips. The proposed method is verified and the results show that the MAPEs of the OD estimation are lower than 19%. In [20], a vehicle trajectory extraction algorithm based on license plate recognition data is proposed. The license plate and timestamps are used for the establishment of trip chain. Aiming at the data loss problem when detecting a vehicle license plate, the K shortest path algorithm and gray relational analysis are further used for trip chain compensation. The research focuses on extracting the vehicle trajectory and the prediction of future driving state is not studied. In [21], authors propose a vehicle trajectory reconstruction method based on license plate data. In the method, travel time threshold is used to obtain a single travel chain and the similarity of the ideal solution and depth first search method are used to build a vehicle trajectory reconstruction model. It can effectively solve the problem of incomplete license plate number data. However, the related research mainly focuses on the macroscopic trajectory modeling and OD analysis and is seldom concerned with the microscopic real-time vehicle trajectory prediction.

## 3. Methods

### 3.1. Trip Chain Building Based on Vehicle License Plate Information Obtained from Video-Imaging Detectors

In this section, we introduce the original data collected by the video-imaging detectors, and establish the corresponding mathematical model based on the actual road network. Meanwhile, using the Dijkstra algorithm, the missing data is supplemented and the travel chain is divided according to the time-cost matrixes.

#### 3.1.1. Preparations for the Trip Chain Building

The whole urban road network consists of intersections and sections. Based on graph theory, the whole road network can be represented by a binary group composed of nodes and edges, as shown in Equation (1).

(1) G=(V,E)

In the binary group, V denotes the set of intersections and E denotes the set of sections. V and E are expressed by Equations (2) and (3) respectively.

(2) $$V=\{v_{1},\cdots ,v_{i},\cdots ,v_{M}\}$$

(3) $$E=\{<v_{i},v_{j}>\}\;i,j\in M$$

where M is the number of intersections in the road network. In Equation (3), $<v_{i},v_{j}>$ denotes that there is a road section between the *i*-th and the *j*-th intersection, i.e., the two intersections are directly connected.

Considering the directions of the sections and the distance between two adjacent intersections, we use the distance and the travelling time to represent the weights of the edge, and the cost matrixes are shown by Equations (4) and (5).

(4) $$Dis=\left[\begin{array}{ccccc} d_{1,1} & \cdots & d_{1,j} & \cdots & d_{1,M}\\ \vdots & \vdots & \vdots \\ d_{i,1} & \cdots & d_{i,j} & \cdots & d_{i,M}\\ \vdots & \vdots & \vdots \\ d_{M,1} & \cdots & d_{M,j} & \cdots & d_{M,M} \end{array}\right]$$

(5) $$Tra=\left[\begin{array}{ccccc} tr_{1,1} & \cdots & tr_{1,j} & \cdots & tr_{1,M}\\ \vdots & \vdots & \vdots \\ tr_{i,1} & \cdots & tr_{i,j} & \cdots & tr_{i,M}\\ \vdots & \vdots & \vdots \\ tr_{M,1} & \cdots & tr_{M,j} & \cdots & tr_{M,M} \end{array}\right]$$

For the cost matrixes, $d_{i,j}$ and $tr_{i,j}$ denote the distance and travelling time, respectively, from the *i*-th intersection to the *j*-th intersection when they are directly connected as given in Equation (3). If the *i*-th intersection to the *j*-th intersection are not directly connected or i=j, $d_{i,j}$ and $tr_{i,j}$ are assigned ∞. In this paper, the travelling time is calculated by vehicle license plate data collected by video-imaging detectors referring to [22,23,24].

Electronic video detectors deployed at the intersection can collect the driving states of a passing vehicle, including the vehicle license plate, detecting time, lane number, vehicle type, body color and others. Moreover, each video detector has basic installation information, for example, the position (longitude and latitude) where the device located, the unique ID of the device, direction of the intersection which it detects, correlations about the intersections and lane. When a single vehicle is on a trip, it will be detected by a series of video detectors on the road and a set of driving states will be formed, expressed as:

(6) $$Ts=\{S_{i}\},i=1,\cdots ,N$$

where *N* is the number of samples during the whole trip. Each sample in the series is presented by Equation (7).

(7) $$S_{i}=(t_{i},u_{i},g_{i},v_{i},h_{i},l_{i},v{’}_{i})$$

In Equation (7), the meaning of each field is explained as follows: 

$t_{i}$ is the detection time. 

$u_{i}$ is the unique ID of the video detector.

$g_{i}$ is the position where the video detector is located. It is expressed by the longitude and latitude.

$v_{i}$ is the intersection where the video detector locates.

$h_{i}$ is the approach direction information of the intersection. In this paper, the direction code is numbered clockwise from a certain approach.

$l_{i}$ is the lane information of the approach. In this paper, the lane code is numbered from inside lane to outside.

$v{’}_{i}$ is the downstream intersection of the current lane. It is acquired by the connectivity of adjacent intersections and channelization.

#### 3.1.2. Trip Chain Optimization and Division Based on Vehicle License Plate

In Equation (6), when all the samples are sorted over time by detection data, the series represents a whole trip chain in the sampling time period. In this section, the whole trip chain is firstly optimized and verified. Furthermore, it is divided into sub-trip chains based on the time interval feature of adjacent samples.

**Figure 1:**
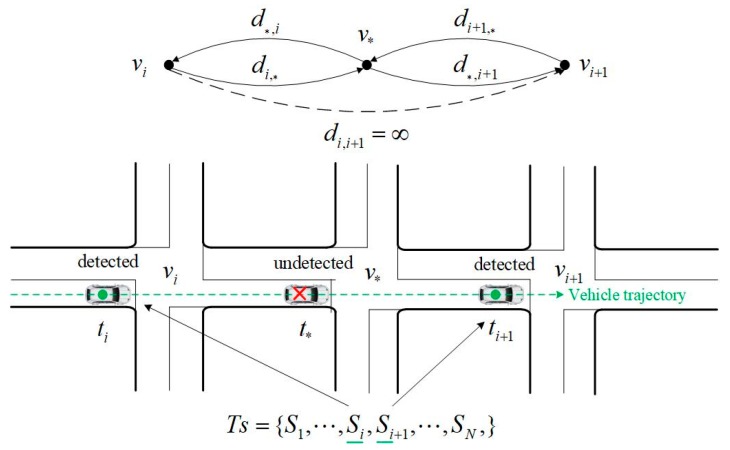
Presentation of missing data and the topology.

In actual applications, some intersections are not installed with video devices or those installed devices may be damaged. Even though the devices work normally, there are still missing detections or errors in detection of the vehicle license plate with a certain probability caused by the poor lighting condition, the performance of license plates recognition algorithm, and other reasons. Hence, the trip chain acquired by the original data of vehicle license plate is not consecutive in general. For some adjacent samples, the two intersections where video devices are located are not directly connected in the road network graph, as shown in Figure 1.

For any two adjacent samples $S_{i}$ and $S_{i+1}$ in Ts, when there is an undetected intersection between them, the values in the cost matrix presented in Equation (4) or (5) should be equal to ∞, that is:

(8) $$d_{v_{i},v_{i+1}}=\infty \;\mathrm{or}\;tr_{v_{i},v_{i+1}}=\infty$$

**Figure 2:**
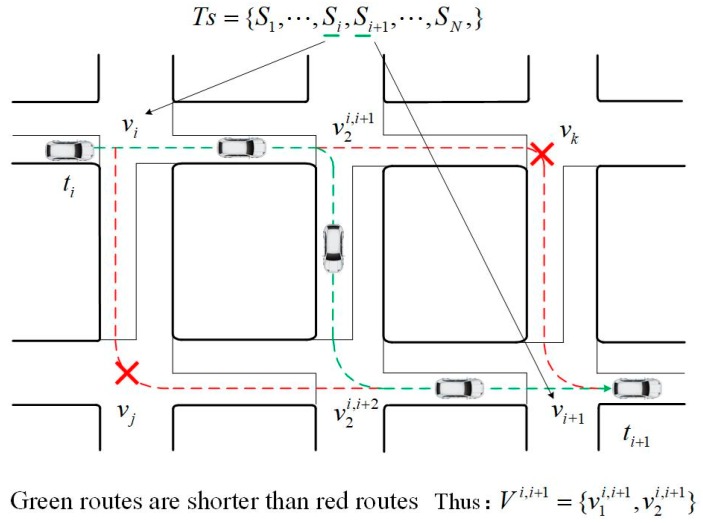
Presentation of intersection series after compensation.

In order to obtain a complete trip chain for further vehicle driving behavior analysis, the data of the undetected intersection should be compensated when there are missing detections between $S_{i}$ and $S_{i+1}$. Suppose that the vehicle drives following the shortest path, the Dijkstra algorithm is used to compensate the trip chain where the two intersections $v_{i}$ and $v_{i+1}$ are taken as the origin and destination, respectively. In the road network graph, the compensating intersections series is described by Equation (9) and the situation is shown by Figure 2.

(9) $$V^{i,i+1}=\{v_{1}^{i,i+1},\cdots ,v_{k}^{i,i+1},\cdots ,v_{N_{c}}^{i,i+1}\}$$

where $v_{k}^{i,i+1}$ denotes the *k*-th intersection between $v_{i}$ and $v_{i+1}$ in the trip chain. $N_{c}$ is the total number of compensating intersections.

After obtaining the compensating intersections, fields such as position, approach direction, lane information and downstream intersection can all be acquired based on the connectivity of adjacent intersections in the road network graph and channelization in actual scenario. 

Considering the calculation error and randomness of the vehicle driving features, we take $\lambda_{i,j}•tr_{i,j}$ as the upper limit of travel time from intersection i to intersection j, where $\lambda_{i,j}$ is the amplification coefficient. If the actual travelling time for a single vehicle is bigger than $\lambda_{i,j}•tr_{i,j}$, it is identified that the vehicle stops between the *i*-th and *j*-th intersection, and the trip chain should be cut off at that place. Referring to this principle, the effectiveness of the compensating nodes is judged as follows:

When the actual travelling time is bigger than sum of upper thresholds of the compensating sections between $v_{i}$ and $v_{i+1}$, as described by Equation (10):

(10) $$\lambda_{v_{k}^{i,i+1},v_{k+1}^{i,i+1}}\sum_{k=1}^{N_{c}}tr_{v_{k}^{i,i+1},v_{k+1}^{i,i+1}}<t_{i+1}-t_{i}$$

The compensating intersections series presented in Equation (9) is ineffective. Under this condition, $v_{i}$ is set as the destination for the former trip chain, and $v_{i+1}$ is set as a new origin for a new trip chain.

Otherwise,

(11) $$\lambda_{i,i+1}\sum_{k=1}^{N_{c}}tr_{v_{k}^{i,i+1},v_{k+1}^{i,i+1}}\geq t_{i+1}-t_{i}$$

The compensating intersections series presented in Equation (9) is effective and the detection time is calculated by Equation (12):

(12) $$t_{v_{k}^{i,i+1}}=t_{i}+\sum_{j=1}^{k}\frac{tr_{v_{j}^{i,i+1}}}{\sum_{l=1}^{N_{c}}tr_{v_{l}^{i,i+1}}}(t_{i+1}-t_{i})$$

By the aforementioned operations, all necessary fields for the compensating samples of a trip chain can be acquired. Since the compensation strategy is proposed based on the assumption that the vehicle drives following the shortest path, there may be some departures with the actual trajectory. To confirm that the compensating samples are accurate enough, we further propose a verification and optimization scheme based on the turning state and downstream intersection. After compensation, the new trip chain can be presented by Equation (13).

(13) $$Ts=\{S_{1},\cdots ,S_{i},S_{1}^{i,i+1},\cdots ,S_{k}^{i,i+1},\cdots ,S_{N_{c}}^{i,i+1},S_{i+1},\cdots ,S_{N}{\}}_{1\times (N+N_{c})}$$

**Figure 3:**
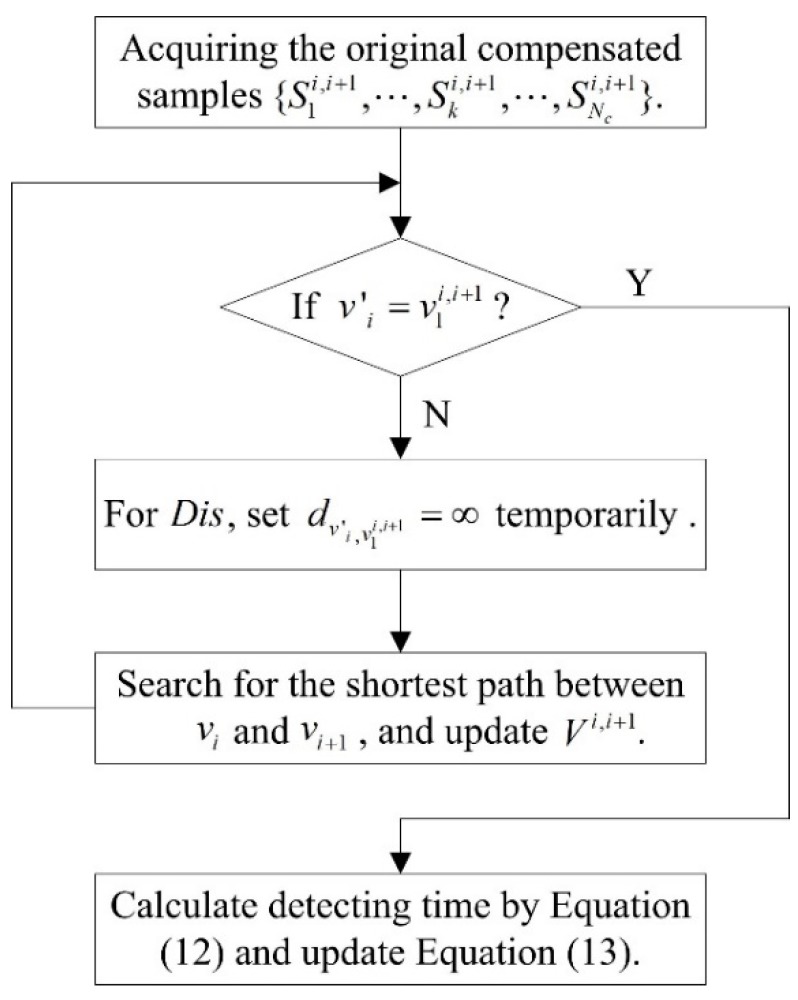
Flowchart of re-compensation algorithm.

In Equation (13), the next sample of $S_{i}$ is $S_{1}^{i,i+1}$. If the downstream intersection $v{’}_{i}$ in $S_{i}$ is not in accordance with the $v_{1}^{i,i+1}$ in $S_{1}^{i,i+1}$, the acquired $N_{c}$ samples are incorrect and should be re-compensated. The re-compensation algorithm flowchart is presented in Figure 3.

For simplicity, the whole trip chain presented by Equation (13) is further expressed by a general form, as shown in Equation (14).

(14) $$Ts=\{S_{1},\cdots ,S_{i},\cdots ,S_{N+N_{c}}\}$$

In the actual scenario, the whole trip consists of one or more sub-trip chains, where each sub-trip chain denotes a complete trip from the origin to destination. The detection time interval of any adjacent samples in Equation (14) denotes the travelling time of the vehicle in the section between $v_{i}$ and $v_{i+1}$. When the time interval is bigger than a certain threshold, it implies that the vehicle finishes the trip at some place between $v_{i}$ and $v_{i+1}$. Under this condition, $v_{i}$ and $v_{i+1}$ belong to different sub-trip chains. Similarly, we take $\lambda_{i,i+1}\cdot tr_{i,i+1}$ as the threshold to divide the trip chain. For the series shown in Equation (14), the detecting time interval of adjacent samples is calculated in order.

(15) $$t_{i+1}-t_{i}>\lambda_{i,i+1}\cdot tr_{i,i+1}$$

The trip chain is divided into two sub-trip chains, in which $v_{i}$ is the destination of the former sub-trip chain, and $v_{i+1}$ is the origin of the following sub-trip chain, as shown in Equation (16):

(16) $$Ts=\left\{ \begin{array}{l} Ts(1)\\ Ts(2) \end{array}\right.$$

where,

(17) $$Ts(1)=\{S_{1},\cdots ,S_{i}\}$$

(18) $$Ts(2)=\{S_{i+1},\cdots ,S_{N+Nc}\}$$

Because of the large data coverage time range, the number of vehicle trips is often greater than two. Therefore, according to the above method, the travel chain can be divided into T sub-trip chains, as shown by Equation (19).

(19) $$Ts=\left\{ \begin{array}{l} Ts(1)\\ \cdots \\ Ts(j)\\ \cdots \\ Ts(N_{T}) \end{array}\right.$$

where $N_{T}$ is the number of sub-trip chains of the vehicle in the sampling time period.

### 3.2. Vehicle Trajectory Prediction Model Based on Turning State Transition Matrix

The series of intersections in the trip chain contains the turning information when a vehicle passes a certain intersection. The turning state transition matrix denotes the probability matrix for which direction a vehicle may take. Considering that the series of intersections for the *j*-th sub-trip chain are denoted by Equation (20):

(20) $$V^{j}=\{v_{1}^{j},\cdots ,v_{i}^{j},\cdots ,v_{N_{j}}^{j}\}$$

Referring to the series presented in Equation (20), it is easy to acquire the downstream intersection of each node in the *j*-th sub-trip chain when the vehicle is driving on the road. In the *k*-th intersection, assuming that there are $N_{a}$ approaches and $N_{e}$ exits with the associated downstream intersections denoted as $\{v_{k}^{’}(1),\cdots ,v_{k}^{’}(N_{e})\}$. The turning state of the case vehicle at a certain intersection can be described by Equation (21).

(21) $$B^{j}={\left[\begin{array}{ccc} b_{a_{1},e_{1}}^{j} & \cdots & b_{a_{1},e_{N_{e}}}^{j}\\ \vdots & \ddots & \vdots \\ b_{a_{N_{a}},e_{1}}^{j} & \cdots & b_{a_{N_{a}},e_{N_{e}}}^{j} \end{array}\right]}_{N_{a}\times N_{e}}$$

In Equation (21), $b_{a_{m},e_{n}}$ denotes the turning relationship when a vehicle drives passing the intersection. When the vehicle enters the intersection from the *m*-th approach and leaves from the *n*-th exit, then,

(22) $$b_{a_{m},e_{n}}=1$$

Otherwise,

(23) $$b_{a_{m},e_{n}}=0$$

For the *j*-th sub-trip chain acquired by Equation (19), the turning relationship can be obtained by the series of intersections and the turning state of the case vehicle (Equation (21)) is established, denoted as $B^{j}$. In extended time, the case vehicle passes the same intersection for many times. Hence, the total turning state of the vehicle at a certain intersection can be calculated by the sum of all the turning state matrixes. For a case vehicle, suppose that there are $N_{T}$ sub-trip chains passing through the *i*-th intersection, the total turning states of the vehicle can be calculated by Equation (24):

(24) $$B_{i}=\sum_{j=1}^{N_{T}}B^{j}={\left[\begin{array}{ccc} \sum b_{a_{1},e_{1}}^{j} & \cdots & \sum b_{a_{1},e_{N_{e}}}^{j}\\ \vdots & \ddots & \vdots \\ \sum b_{a_{N_{a}},e_{1}}^{j} & \cdots & \sum b_{a_{N_{a}},e_{N_{e}}}^{j} \end{array}\right]}_{N_{a}\times N_{e}}$$

In addition, the turning state transition matrix is acquired by Equation (25):

(25) $$Pr_{i}=B_{i}/N_{T}={\left[\begin{array}{ccc} \sum b_{a_{1},e_{1}}^{j}/N_{T} & \cdots & \sum b_{a_{1},e_{N_{e}}}^{j}/N_{T}\\ \vdots & \ddots & \vdots \\ \sum b_{a_{N_{a}},e_{1}}^{j}/N_{T} & \cdots & \sum b_{a_{N_{a}},e_{N_{e}}}^{j}/N_{T} \end{array}\right]}_{N_{a}\times N_{e}}$$

In Equation (25), $\sum b_{a_{m},e_{n}}^{j}/N_{T}$ denotes the probability when the vehicle chooses the *n*-th exits from the *m*-th approach. For each row of the matrix shown in Equation (25), the following constraints should be satisfied:

(26) $$\sum_{n=1}^{N_{e}}\sum b_{a_{m},e_{n}}^{j}/N_{T}=1$$

Equation (26) implies that, in case the vehicle drives in an intersection from a certain approach, it must go out from one of the exits. However, for some intersections, there may be no effective trip chain, i.e, the case vehicle does not pass the intersection during experimentation. When this happens, the turning state probability for each exit is assigned an equal average probability value, as shown in Equation (27):

(27) $$\sum b_{a_{m},e_{n}}^{j}/N_{T}=1/N_{e}$$

When a vehicle enters the intersection from the *m*-th approach, it will go to the *n*-th downstream intersection from the *n*-th exit at a probability of $\sum b_{a_{m},e_{n}}^{j}/N_{T}$. Hence, the one-step prediction probability of the vehicle for the next intersection $v{’}_{i}$ is calculated by Equation (28).

(28) $$P_{i+1}=O_{i}\cdot Pr_{i}={\left[o_{a_{1}}^{i},\cdots ,o_{a_{N_{a}}}^{i}\right]}_{1\times N_{a}}\cdot {\left[\begin{array}{ccc} \sum b_{a_{1},e_{1}}^{j}/N_{T} & \cdots & \sum b_{a_{1},e_{N_{e}}}^{j}/N_{T}\\ \vdots & \ddots & \vdots \\ \sum b_{a_{N_{a}},e_{1}}^{j}/N_{T} & \cdots & \sum b_{a_{N_{a}},e_{N_{e}}}^{j}/N_{T} \end{array}\right]}_{N_{a}\times N_{e}}$$

In Equation (28), $O_{i}$ is the original state of the vehicle. When the vehicle is originally detected at the *m*-th approach:

(29) $$o_{a_{m}}^{i}=1$$

For other approaches,

(30) $$o_{a_{1},\cdots ,a_{m-1},a_{m+1},\cdots ,a_{N_{a}}}^{i}=0$$

From Equation (28), the turning probabilities of the vehicle to the downstream intersections are acquired, expressed by Equation (31):

(31) $$P_{i+1}=[p_{e_{1}}^{i+1},\cdots ,p_{e_{N_{e}}}^{i+1}]$$

**Figure 4:**
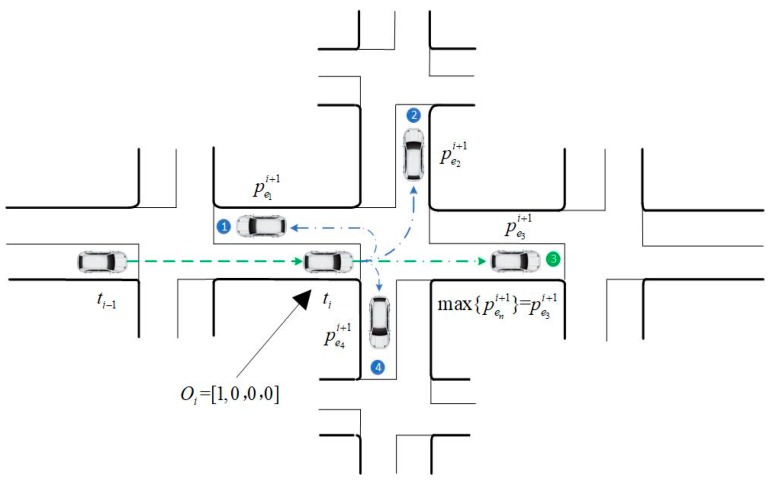
One step trajectory prediction.

Based on the analysis above, the one-step predicted intersection which the vehicle will pass through is the downstream intersection corresponding to the maximum probability $\max\{p_{e_{n}}^{i+1}\}$. The one-step prediction method can be described by Figure 4.

The *k*-step prediction probability of the vehicle for the next *k* intersections is calculated by Equation (32).

(32) $$P_{i+k}=O_{i+k-1}\cdot Pr_{i+k-1}$$

In Equation (32), $O_{i+k-1}$ is the original probability state of $v_{i+k-1}$. Referring to the directly connected relationship with the upstream intersection $v_{i+k-2}$, assuming that the vehicle comes from the *m*-th approach of $v_{i+k-1}$, $o_{a_{m}}$ is valued as the turning probability based on the upstream intersection $v_{i+k-2}$. For other approaches,

(33) $$o_{a_{1},\cdots ,a_{m-1},a_{m+1},\cdots ,a_{N_{a}}}^{i+k-1}=0$$

Similarly, the *k*-step predicted intersection is the *k*-th downstream intersection corresponding to the maximum probability $\max\{p_{e_{n}}^{i+k}\}$.

**Figure 5:**
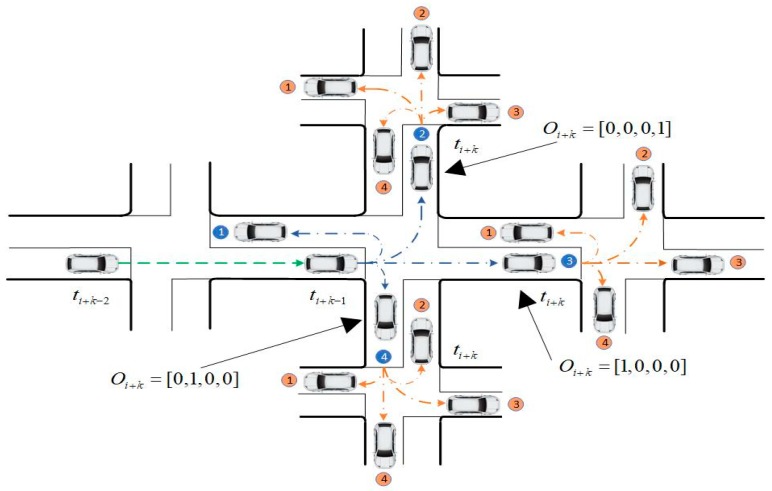
K-step trajectory prediction.

The *k*-step prediction method can be described by Figure 5.

## 4. Experiments and Discussion

**Figure 6:**
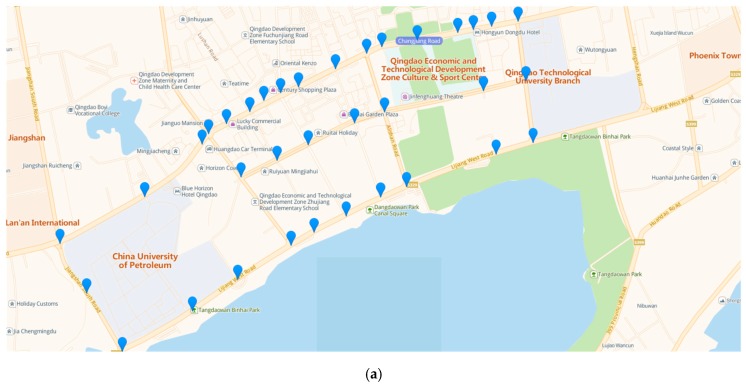

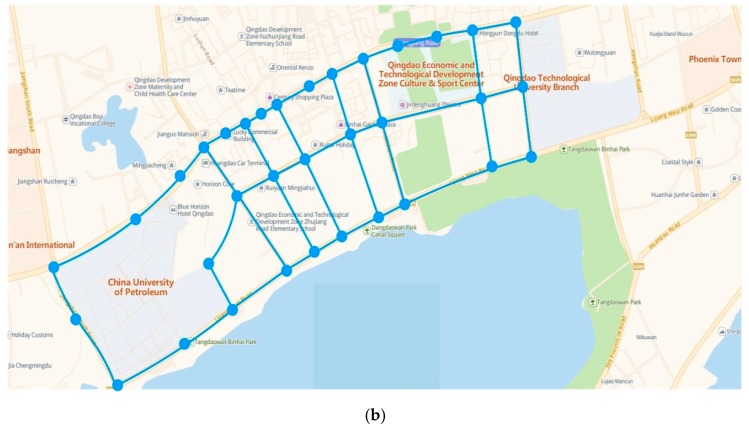
The positions of video-imaging cameras and road network topology in the case study. (**a**): Distribution of video-imaging cameras. (**b**): Road topology.

In this section, a regional road network in Qingdao, China, is selected for the case study. In the network, there are 27 intersections, 40 sections and 35 positions deploying with video-imaging cameras, as shown in Figure 6.

The original vehicle license plate data sample is acquired from the video-imaging detectors in actual traffic scenario. The proposed method is evaluated based on actual historical video-imaging data for the duration of one month.

### 4.1. Results of Trip Chain Building and Compensation

In the proposed method, travel time threshold between adjacent intersections is highly necessary for dividing the trip chain into different sub-trip chains. Since the traffic states are different at different time periods per day, the threshold should be calibrated according to the traffic variation. In this section, we take the morning and evening rush hours as examples to present the amplification coefficient $\lambda_{i,j}$ calibration progress.

**Figure 7:**
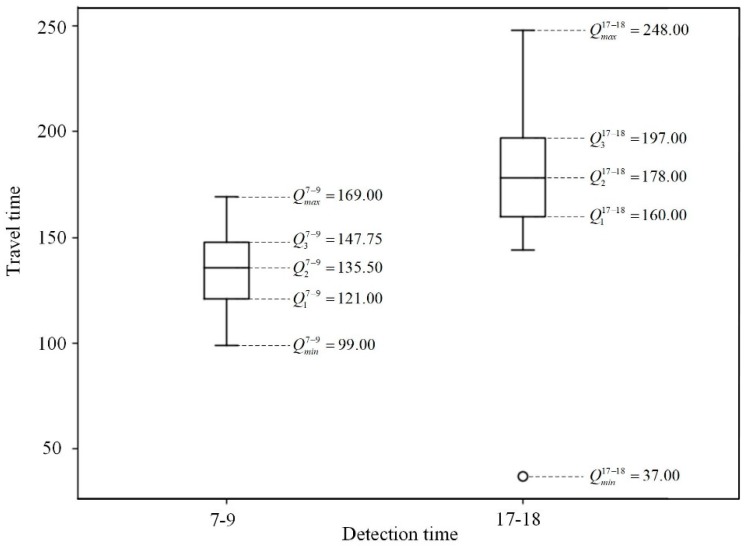
An example of travelling time value distribution of the case section.

Considering the section in Figure 6b as an example, the samples at 7:00–9:00 AM for morning rush hours and 17:00–18:00 for evening rush hours in the original dataset in one month are extracted and analyzed, and the statistics of travelling time for all vehicles are presented in Figure 7. 

From Figure 7, it is evident that the traffic flow of the case section has typical tidal feature since the travelling time values in evening rush hours are much higher than the mornings. However, the travel time values are clustered in major regions referring to different time periods. If the threshold is set too small, some normal sub-trip chains will be over-segmented and much useful information will be lost for the establishment of the turning state transition matrix. If the threshold is too big, two sub-trip chains will be considered as one, inducing a misjudgment of the vehicle travelling state at that the joint points. In order to avoid this, the amplification coefficient $\lambda_{i,j}$ is calculated by the ratio of the upper value and the average travelling time in Equation (5) after excluding data outliers, as shown in Equation (34).

(34) $$\lambda_{i,j}=Q_{max}/Tr_{i,j}$$

After acquiring the trip chains of a target vehicle, missing data points are compensated by the method proposed in Section 3. In order to evaluate the performance of the proposed trip chain compensation method, part of consecutive sampling nodes are selected and removed artificially from a whole trip chain. In this paper, at most 5 consecutive sampling nodes are compensated for. Based on the original trip chain shown in Equation (6), 1 to 5 consecutive sampling nodes are removed respectively to obtain sample sequences for compensation, as shown in Equation (35).

(35) $$Ts_{con}=\{S_{1},\cdots ,S_{i-1},S_{i+n_{con}},\cdots ,S_{N}\},i=2,3,\cdots ,N-n_{con}$$

In Equation (35), The total number of cases is $N-n_{con}$, and the number of consecutive nodes for compensation is $n_{con}$.

**Figure 8:**
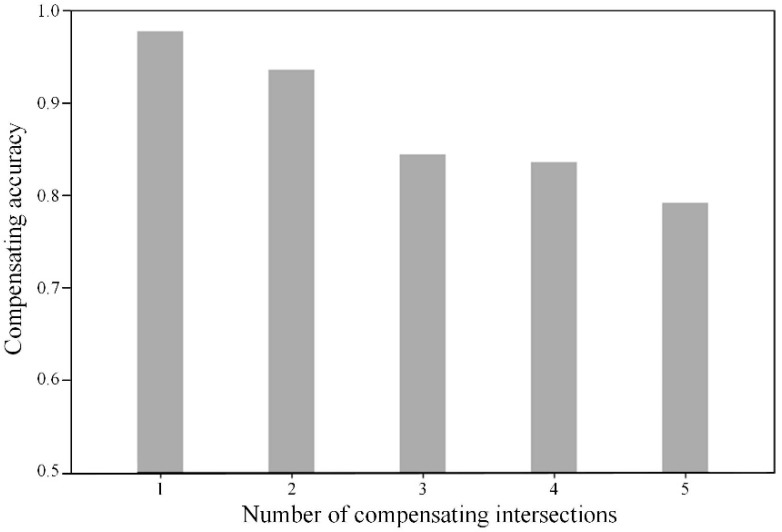
Compensating accuracy under different cases.

Using the method proposed in Section 3.2, the removed nodes in Equation (35) are compensated. To assess the performance of the compensation method quantitatively, the compensation accuracy is proposed. It is calculated by the ratio of the number of correct nodes after compensation to the total number of nodes for compensation. The compensating accuracy under different cases is shown in Figure 8.

From Figure 8, it is evident that the proposed method presents a significant performance in the compensating missing nodes. All the cases are with a high accuracy of more than 80%. Moreover, the accuracy presents a declining trend with the increase of the number of nodes for compensation. This is because when several consecutive sampling nodes are missing, there will be more possible trajectories for the vehicle in the undetected region. The Dijkstra method may not perform very well in a large and complex road network.

### 4.2. Trajectory Prediction Results and Analysis

**Figure 9:**
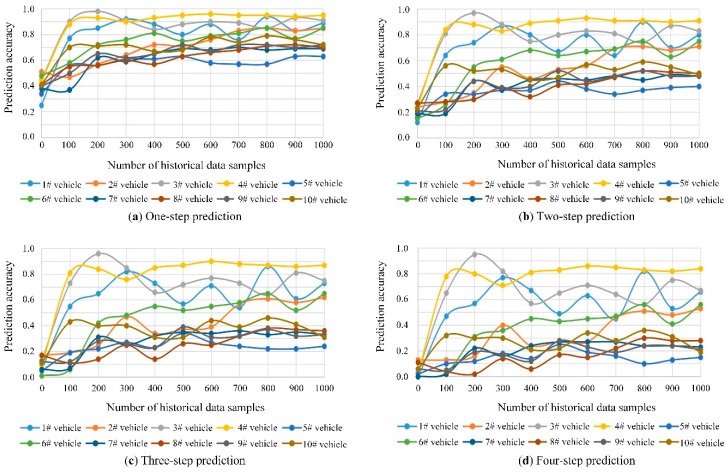
One to four-step trajectory prediction results of case vehicles.

Among all vehicles in the original data sample, a section of the vehicles are selected for the verification of the performance of trajectory prediction. In this paper, all the trip chains of the case vehicles are acquired from the original data. Moreover, part of the historical trip chains are used for training the turning state transition matrix and the remaining trip chains are used for testing the accuracy of the trajectory prediction results. For one to four-step prediction, the results of 10 case vehicles are presented in Figure 9.

**Figure 10:**
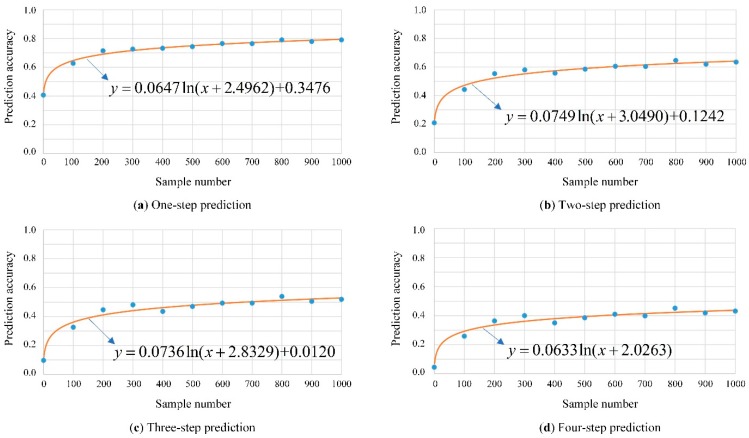
Average prediction accuracy and the fitting results for different prediction steps.

In Figure 9, it is evident that, the accuracy varies significantly among different vehicles. As shown in Figure 9, 1#, 3# and 4# vehicles present a much higher prediction accuracy than others for one to four-step trajectory. This presentation is mainly caused by the regularity of vehicle driving characteristics. For vehicle trajectories that are relatively regular, such as the trajectories created by the commuters to and from work in each working day, the accuracy presents much high and stable values, while for the random travelling trajectories, such as the trajectories from taxies, the accuracy is relatively low. For example, the 5# vehicle presents a low prediction accuracy and large fluctuation with the gradual increase of training data. In order to show the results more clearly, the average prediction accuracy for testing vehicles together with the fitting results are further presented in Figure 10. According to the variation of the accuracy values, the logarithmic function is applied for the fitting, as shown in Equation (36).

(36) y=aln(x+b)+c

In Figure 10, with the increase of the amount of training data, the accuracy presents a rising trend. More training data contains more information about the trip chains so that the turning state transition matrix can describe the travelling characteristics more accurately. In the case analysis, vehicles can reach an average accuracy of 0.72 for one-step prediction on the basis that there are more than 200 training data samples. Hence, the proposed method presents a better performance in trajectory prediction. Moreover, the accuracy presents an overall downward trend with the increase of number of prediction steps. The maximum accuracy is about 0.80, 0.63, 0.51 and 0.43 for one-step, two-step, three-step and for four-step trajectory prediction, respectively. The reason is that there are more cases for the vehicle to choose the following intersections with the increase of the number of prediction steps. As the trajectory becomes more unpredictable, the accuracy declines.

## 5. Conclusions and Future Work

This paper proposes a vehicle trajectory prediction algorithm based on license plate data collected from video-imaging detectors. In order to obtain more complete vehicle travel information, we use the Dijkstra algorithm for data compensation. The driving characteristics are described by the turning state transition matrix which is acquired by the historical trip chains based on the time series of license plate data. Based on the turning state transition matrix, we make a multi-step prediction for specific vehicles. The experimental results show that, although the performance of trajectory prediction for different vehicles varies significantly, the proposed vehicle trajectory prediction algorithm has high average accuracy at the expense of a simple calculation, especially for one-step prediction. Compared with the traditional schemes, the proposed method fully exploits the potential value of existing data and without any extra investment needed. This is really beneficial for urban traffic feature analysis and traffic management. 

In this paper, the vehicle license plate data obtained from video-imaging detectors is the unique input of the proposed method. A high-quality license plate data set is the prerequisite for the implementation of the method. Some subtle errors in the original data, such as timestamp error, detector positioning error and others, should be eliminated. Hence in actual applications, a sophisticated data pre-processing scheme is indispensable. 

Future research mainly focuses on two aspects. Firstly, the proposed method can be verified using a license plate data set of 10 vehicles in one month. In order to acquire more precise conclusions, the data sample should be further expanded. Secondly, according to the general understanding, the driving characteristics of a section of vehicles in an urban environment is time-sensitive to some extent. Hence, an analysis of the sensitivity of historical data to the prediction accuracy will be carried out.
